# Dose-Finding Based on Bivariate Efficacy-Toxicity Outcome Using Archimedean Copula

**DOI:** 10.1371/journal.pone.0078805

**Published:** 2013-11-12

**Authors:** Yuxi Tao, Junlin Liu, Zhihui Li, Jinguan Lin, Tao Lu, Fangrong Yan

**Affiliations:** 1 State Key Laboratory of Natural Medicines, China Pharmaceutical University, Nanjing, China; 2 Research Center of Biostatistics and Computational Pharmacy, China Pharmaceutical University, Nanjing, China; 3 Department of Mathematics, China Pharmaceutical University, Nanjing, China; 4 Department of Mathematics, Southeast University, Nanjing, China; Queen’s University Belfast, United Kingdom

## Abstract

In dose-finding clinical study, it is common that multiple endpoints are of interest. For instance, efficacy and toxicity endpoints are both primary in clinical trials. In this article, we propose a joint model for correlated efficacy-toxicity outcome constructed with Archimedean Copula, and extend the continual reassessment method (CRM) to a bivariate trial design in which the optimal dose for phase III is based on both efficacy and toxicity. Specially, considering numerous cases that continuous and discrete outcomes are observed in drug study, we will extend our joint model to mixed correlated outcomes. We demonstrate through simulations that our algorithm based on Archimedean Copula model has excellent operating characteristics.

## Introduction

The fundamental objective of drug development is to find a dose, or dose range, of a new drug. It is believed by many that the high attrition rate currently observed in phase III is largely driven by inadequate dose selection [1]. In most common dose-finding designs and methods, Phase I trials are designed to study the safety of the test drug, and maximum tolerable dose(MTD) is determined only based on toxicity. Efficacy outcomes are observed but not used. In Phase II trials, efficacy of the test drug is studied [2], [3]. However, under a variety of circumstances, adverse events may lead to the early termination of drug development before efficacy outcome is observed, and there is a probable correlation between efficacy and toxicity. Besides that, valuable information may be lost if the efficacy outcome is ignored in Phase I.

Therefore, it is necessary to establish joint model for toxicity and efficacy to choose optimal dose and analyze their correlation. A number of joint modeling strategies have been studied in the literature [4], [5]. In the context of an HIV trial, O’Quigley, Hughes, and Fenton [6] proposed model 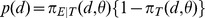 to describe the relationship of dose and success rate. In 1998, Peter F.Thall and Kathy E.Russell [7] combined the toxicity and efficacy into a single trinomial variable, then used a proportional odds regression (PO) model for the relationship of dose and the single trinomial variable. Afterwards, Wei Zhang, Daniel J.Sargent [8] adopted continuation-ratio(CR) model instead of PO model. In 2002, Braun [9] provided a bivariate distribution for correlated binary outcomes. This distribution contains a parameter 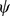 to represent the association between two outcomes. In 2005, Vladmir Dragalin, Valerii Fedorov [10] modeled the distribution of bivariate binary endpoint using either Gumbel bivariate logistic regression or Cox bivariate binary model. Moreover, Valerii Fedorov [11], [12], [13] provided a bivariate probit model in 2007. Most joint modeling methods above are only suitable for binary endpoint, and model the relationship of dose and probability. However, continuous outcomes are also often observed in clinical trials. For example, shrinkage in a solid tumor, changes in blood pressure, or changes of glomerular filtration rate. In 2010, Aiyang Tao [14] studied bivariate continuous outcomes by bivariate normal distribution, and use AMCP-MOD method which is a fixed design with pre-determined randomization strategy to find optimal dose for phase III.

In our article, we propose Archimedean Copula joint model to evaluate efficacy and toxicity simultaneously. We mainly focus on two cases: bivariate continuous outcome and a mixture of continuous and categorical outcomes. Compared with other joint models, Archimedean Copula joint model has four main advantages: 1) The Copula joint model has no restrictions on probability distributions of efficacy and toxicity. 2) Marginal parameters in Copula joint model for both efficacy and toxicity are meaningful; 3) Copula joint model has few limitation of data type. 4) Archimedean Copula has explicit joint distribution formula. Bivariate mixed outcome is one of most analytically difficult cases, because they do not follow an obvious multivariate distribution. Based on this nice property of Archimedean Copula, we can easily structure the joint distribution via marginal distribution of continuous outcome and the conditional distribution of discrete outcome. In addition, Archimedean Copula is not restricted to radial symmetry. Take three popular Archimedean Copulas as examples. The Clayton Copula is an asymmetric Archimedean Copula, exhibiting greater dependence in the negative tail than in the positive. The Frank Copula is a symmetric Archimedean Copula. The Gumbel Copula is an asymmetric Archimedean Copula, exhibiting greater dependence in the positive tail than in the negative. According to different situations, we can choose corresponding Archimedean Copula. In fact, Archimedean Copula has another merit that they allow modeling dependence in arbitrary high dimensions with only one parameter. However, we mainly focus on bivariate efficacy –toxicity outcome in this article, so this merit is not obvious.

Continuous reassessment method(CRM) [15]is a popular algorithm in Phase I cancer clinical trials which is proposed to select maximum tolerated dose(MTD) [16], [17], [18].It is also an adaptive design which means design is guided by examination of the accumulated data at an interim point in the clinical trial. It aims to (1) keep to a minimum number of patients treated at unacceptable high toxic dose levels. (2) keep to a minimum number of undertreated patients (3) respond quickly to errors in initial estimates, rapidly escalating in the absence of indication of drug activity and rapidly de-escalating in the presence of unacceptable high levels of observed toxicity (4) come to an early closure if there is no appropriate dose. According to its good character, we adopt CRM approach and extend it to bivariate trials to select the optimal dose for phase III in our article.

The remainder of the paper is organized as follows. In ‘Methods’ section, we propose Copula joint model for bivariate efficacy-toxicity outcome in detail, and dose-finding design is also discussed here. ‘Results’ section provides simulations to illustrate the performance of proposed model and algorithm. Finally, a conclusion is made in the part of “Discussion”.

### Motivating Example

#### Bivariate continuous outcomes

ACE inhibitors(inhibitors of angiotensin-converting enzyme)are used primarily in treatment of hypertension and heart failure. However, renal impairment is a significant adverse effect of all ACE inhibitors. The reason for this is still unknown.Some suggest that it is associated with their effect on angiotensin II-mediatedd homeostatic functions such as renal blood flow. Renal blood flow may be affected by angiotensin II because it vasoconstricts the efferent arterioles of the glomeruli of the kidney, thereby increasing glomerular filtration rate (GFR). Hence, by reducing angiotensin II levels, ACE inhibitors may reduce GFR, a marker of renal function. In one clinical trial an ACE inhibitor is used to treat hypertension. The efficacy endpoint is the change of sitting blood pressure from baseline. Decreasing GFR is the undesirable effect and the main toxicity measure is the change of GFR from baseline.

#### Bivariate mixed outcomes

Anticoagulants are pivotal agents for the prevention and treatment of thromboembolic disorders. The efficacy of Anticoagulant is to lower the VTE(venous thromboembolism) incidence rate. Unfortunately, such an effect can be accompanied by an increase in major bleeding, especially postoperative, during the treatment. Therefore, when choosing the optimal dose, we should consider the VTE incidence rate and bleeding event simultaneously.

## Methods

### Archimedean Copula for Bivariate Correlated Efficacy-toxicity Outcome

Copula is recently popular strategy for joint modeling in statistical applications. The history of Copula may begin with Frechet [19], who studied the problem in low-dimension. In 1959, Sklar researched in-depth in this respect, by introducing the notion, and the name, of a Copula, and proving the theorem that now bears his name [20]. Sklar’s theorem states that a multivariate cumulative distribution function of n random variables can be written as
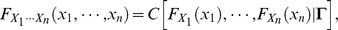
where 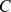 is a Copula, and 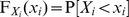.

In our study, Let 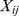 and 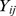 respectively denote the efficacy and toxicity outcomes obtained from 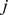 subject within fixed dose group 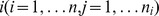. For each dose *d_i_*, we assume 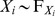, and 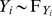. The marginal distributions can be arbitrary continuous distributions. To specify the marginal models, let 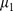 and 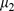 be suitable link models chosen from common candidate models like Emax, Exponential, logit, etc.
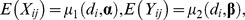
here, 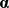 and 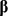 are marginal model parameter vectors.

For Archimedean Copula, the Copula function 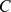 is defined as:
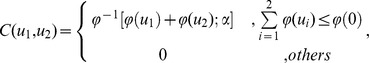
where 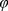 is the so called generator, 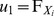, 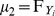, and 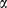 is the association parameter measuring dependence between efficacy and toxicity. Some popular Archimedean Copulas, their corresponding generators and association parameters are listed in Table 1.

**Table 1:** Archimedean Copula, generator and association parameter.

Copula	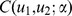	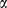	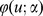
Frank	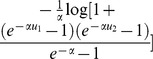	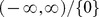	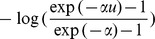
Gumbel	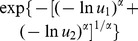	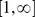	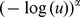
Clayton	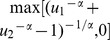	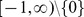	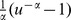

Note that all marginal parameters are inherited from the 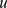 with meaning, and the parameters for association are brought into the Copula parameter 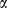. For Frank Copula and Clayton Copula, only if 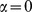, the outcomes are independent, and when 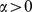 or 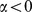, outcomes have positive and negative associations; For Gumbel Copula, when 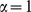, the outcomes are independent, and when 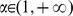, outcomes have positive associations.

When efficacy 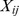 and toxicity 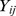 are both continuous outcomes. The joint distribution 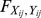 determined by Archimedean Copula takes the form in Table 1, and the density function of Archimedean Copula is given by:
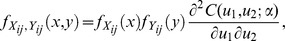
where 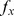 and 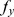 represent corresponding marginal density function.

When the margins appear to be mixed outcomes, we adopt another strategy to build joint model. Assume efficacy 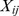 is continuous and toxicity 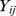 is categorical. Similarly, let 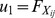 and 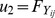, but 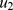 is discrete distribution here. Let
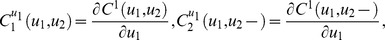
where 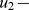 is the left-hand limit of 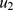. Then, the joint density is given by



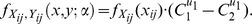



Take Frank Copula for example, the joint distribution 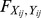 determined by Frank Copula takes the form
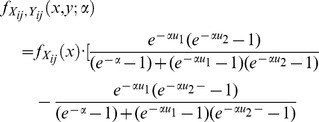



Copula is popular in statistical applications as it allows one not only to conveniently build joint regression model, but also to easily estimate the parameters in joint regression model by using Copula density function. Once we get the Copula joint density function, we can use maximum likelihood estimation, which has come to be quite mature and be integrated in many softwares.

### Decision Rules

#### Dose-finding criteria

After the Copula joint model is established and successfully fitted to data, the optimal dose(s) to be carried into Phase III still remains unresolved. We calculate the minimum effective dose (MED) and the maximum tolerated dose (MTD) first.

Following Ruberg [21], the MED is defined as the smallest dose, which shows a clinically relevant and a statistically significant effect. Let 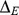 denotes the clinically relevant difference with respect to the smallest dose (often placebo 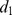). Note that 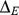 does not depend on the particular dose-response model under consideration, but only depend on the objectives of the guidelines or clinicians. Given a marginal model 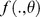 for efficacy, 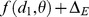 is the clinically relevant effect, and
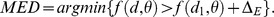



Similar, MTD is defined as the maximum dose which shows clinically acceptable toxicity. Let 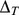 denotes the clinically acceptable difference, that is, that largest toxicity acceptable difference, by which we expect a dose to be not too worse than placebo. A marginal model 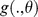 for toxicity is given as follow:
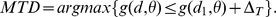



If the interval [MED, MTD] called therapeutic range exists, we can select the final optimal dose based on the following utility function [22]. The utility function is
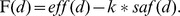



The final dose is determined by maximizing the utility function. Here, 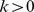 represents the weight for the discounted toxicity from efficacy.

For example, we expect efficacy outcome 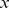 would increase, and toxicity outcome 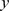 would not decrease heavily after take medicine. Then
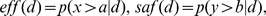
where 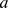 and 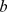 are the desired efficacy threshold and toxicity threshold. In general, a and b are tightened than clinical acceptable threshold to increase the success rates in phase III. Other situations can be solved by changing the operator in above expression.

#### Dose finding design

We adopt CRM approach and extend it to bivariate response to select the optimal dose for phase III in our article. Due to safety and ethics concerns, the up and down design [3] will be used until the first toxicity is observed. The trial is carried out as follows.

1. Treat subjects in cohorts of size c, up to maximum of N subjects. Dose-response (efficacy) model and dose-response (toxicity) model are assumed. Set fixed dose groups.
2. Define the clinically acceptable difference 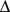 for efficacy and toxicity, and define the desired thresholds for efficacy and toxicity in utility function.
3. The up and down design will be used for the first cohort and will be stopped after the first observed toxicity.
4. Collect the efficacy and toxicity responses to fit joint models, and calculate the initial therapeutic range [MED, MTD].
5. If MTD is smaller than MED, our trail is stop and there is no appropriate dose for phase III. If MTD is larger than MED, fixed dose groups in the Therapeutic range are delivered for next cohort. Then, return to the step4.
6. Once the predetermined fixed sample size is reached, the final dose is selected by utility function.

#### Sample size

An optimal sample size can be derived through simulation. Under a scenario where the efficacy at the selected dose 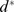 equals the MED, specially, 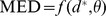. For given sample size N, and interval width 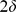, simulations can determine the probability 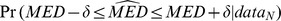. Then we can choose the sample size N based on following formula. Here, target probability is pre-specified as
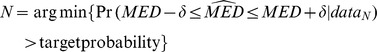



In our following simulations, we set 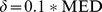, and target probability is 0.7. Note that marginal model for continuous outcome often contains many parameters. When we decide the size of cohort, this point need to be considered.

## Results

### Simulation

We examine the operating characteristics of our algorithm in a study seeking for optimal dose for phase III. We simulate six different scenarios here, allowing the evaluation of the methods under a wide range of scenarios likely to be observed in clinical practice.

#### Example of bivariate continuous outcomes

**Figure 1:**
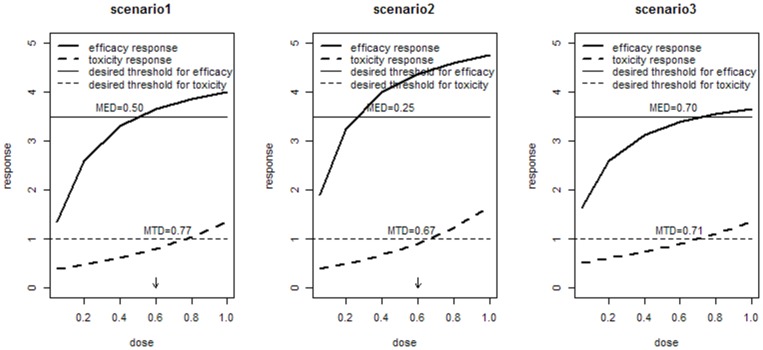
Dose-response curves in first three scenarios.

We examine the operating characteristics of Archimedean Copula in the ACE inhibitors study through simulations. The performance is illustrated under three possible scenarios, which are shown in Figure 1, and the true optimal dose indicated by arrows.

We assume efficacy outcome–the decreased DBP from baseline 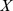 follows normal distribution and Emax model,
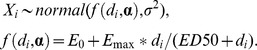



The toxicity outcome–the decreased GFR from baseline 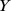 follows exponential distribution and exponential model.
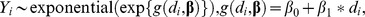
here, 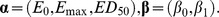


We select Gumbel Copula as our joint model. The modeling approach and dose-finding algorithm can easily extended to other Archimedean Copula models with similar process. We assume association parameter 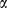 = 1.67 which represents a moderate correlation between two outcomes. The sample size of subjects is 175, and cohort size c = 20/dose. We have a fixed dose group DOSE = 0.05, 0.2, 0.4, 0.6, 0.8. To adequately estimate the operating characteristics of our algorithm, 1000 simulations are conducted for each scenario. The data is generated using the mvdc function in the “copula” library in R software.

To illustrate the advantages of Copula joint model, we compare its performance with Tao’s joint model [14]. Considering that both outcomes in Tao’s joint model must follow normal distribution, we assume toxicity outcome
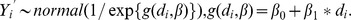



The performance of our algorithm under first three possible scenarios is shown in Table 2. It is obvious that algorithm based on Copula model performs better than Tao’s joint model and separate model, and Tao’s joint model performs worst among them. This shows that when response does not follow normal distribution, Tao’s joint model is not applicable. In scenario1, the algorithm based on Copula model selects correct dose in 59.4% of simulation. Its accuracy rate is lower than Copula in other two scenarios.The basic reason is that the therapy range in secenario1 is narrow. At the beginning of clinical trials, relatively few enrolled subjects will affect the accuracy of therapy range estimation, and then affect the dose selection. In fact, due to the narrow range, 35.2% simulations in scenario 1 are informed there is no dose in therapeutic range. In scenaro2, our algorithm correctly identifies the dose in 810 of the1000 simulations.It is nearly 10 percent higher than separate model and Tao’s joint model. More specifically, in more than 12% simulations, the dose which is only one level below the correct dose is selected in scenario 2. In the scenario 3, there is no suitable dose taken to Phase III in setting, and 89% simulations made the right judge by Copula model. Besides that, we also expect to terminate the study early due to excess toxicity and less subjects are under toxicity. Actually, study indeed ends early, and only 67% subjects enter the trial. Based on Table 2, the average number of subjects given dose under toxicity is 0.256 which seems higher than that in first two scenarios. However, the average number of enrolled subject is only 67%. Therefore, in fact, the average number of subjects under toxicity is as low as first two scenarios.

**Table 2:** Operating characteristics under scenario1–3.

	Copula joint model			Tao’s joint model			Separate model		
scenario	1	2	3	1	2	3	1	2	3
Trials where correct dosefound(%)	0.594	0.810	0.890	0.494	0.703	0.860	0.585	0.705	0.856
Average number subjects givendose under inefficacy(%)	0.349	0.114	0.744	0.373	0.121	0.729	0.356	0.114	0.745
Average number subjects givendose under toxicity (%)	0.138	0.133	0.256	0.164	0.166	0.271	0.141	0.142	0.255

Besides, for security reasons, we use up and down method at the beginning of clinical trial, so there must be some subjects taking dose which is inefficacy. But regardless of the scenario with no efficacy dose, our algorithm can keep to a fewer the number of undertreated subjects. When the therapeutic range is wide, about 11.4% subjects enter the undertreated dose group. As the scope decrease, the number will increase with the maximum of 35% in our simulation. In scenario3, the average number of subjects under inefficacy is up to 74.4%. It is because there is no fixed dose in therapy range. The dose which is efficacy is also toxicity. So enrolled subject takes either toxicity dose or inefficacy dose. In this case, trial ends early under our algorithm, and only 67% subjects enter this clinical trial.

#### Example of bivariate mixed outcomes

**Figure 2:**
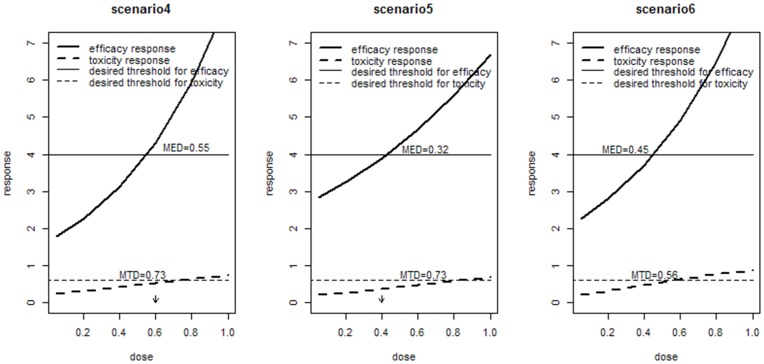
Dose-response curves in last three scenarios.

We assess the operating characteristics of Archimedean Copula in the prevention of VTE trials via simulation studies. The performance is also illustrated under three possible scenarios which are shown in Figure 2, and the true optimal dose indicated by arrows.

The efficacy outcome is the lowered VTE incidence rate 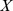, which follows normal distribution and linear model,
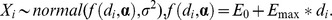



The toxicity outcome is having major bleeding event 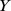 with 1 representing occurrence and 0 representing no occurrence. 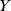 follows the Bernoulli distribution and logistic model.
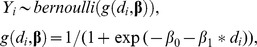
where, 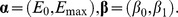


We also select Gumbel Copula as our joint model. Sample size of subjects is 225, and cohort size c = 20/dose. We have a fixed dose group DOSE = 0.05, 0.2, 0.4, 0.6, 0.8. The association parameter 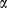 = 20 which represents a highly correlation between two outcomes. To adequately estimate the operating characteristics of our algorithm, 1000 simulations are conducted for each scenario. The performance of Copula model is shown in Table 3.

**Table 3:** Operating characteristics under scenario4–6.

	Copula model			Separate model		
scenario	4	5	6	4	5	6
Trials where correct DOSE found(%)	0.664	0.784	1	0.593	0.745	1
Average number subjects given dose under inefficacy(%)	0.344	0.265	0.987	0.345	0.264	0.974
Average number subjects given dose under toxicity(%)	0.202	0.192	0.013	0.217	0.213	0.026

The performance of our algorithm under last three scenarios is shown in Table 3. Copula model performs better than separate model in these three scenarios. In scenario4, 66.4% simulations select the right dose based on Copula joint model. Similarly, it is the lowest value among last three scenarios because of the narrow therapy range. In scenaro5, Copula model correctly identifies the dose in 784 of the1000 simulations. In scenarios 6, the accuracy rate of dose selection is up close to 1, and only 58.1% subjects enter clinical trial. In addition, in scenarios4–5, the average number of subjects under toxicity is about 20%, and average number of subjects under inefficacy is about 30%.

## Discussion

Assessment of dose-response profiles for efficacy and toxicity outcomes is the key to reliable evaluations of the risk-benefit profile of a drug as well as the selection of final doses to be carried into clinical trials. In this article, we develop Archimedean Copula joint model for bivariate efficacy-toxicity outcome of new drug. We study in detail two situations: one for bivariate continuous variates, and the other for bivariate mixed outcome. Simulation results indicate that Copula joint model provides better design performance than separate model in both cases.

Actually, lots of remaining interesting issues are unresolved. The following directions are considered as important potential study directions in the future. 1) There are many useful Archimedean Copulas. Obviously, if one knows the “right” Copula, the Copula-based regression analysis is more effective. But which one is “right”? In this article, we take the Gumbel as example, but we can use AIC or other model selection method [23] to select optimal Copula model. Actually, we had intended to add model selection into simulation. Weigh the practicability and the amount of calculation, we present the current design. 2) We assume throughout the paper that efficacy and toxicity responses have no relation with covariables; however, many observations in the drug study are affected by covariables, such as age, gender, etc. Extending our methodology to allow for individual characteristics in the data is meaningful. 3) To address different objectives, a number of different dose-finding approaches have been developed. Simulation and comparison of their character based on Copula joint model will be the focus of our study in the future.
